# Fetal Calcifications Are Associated with Chromosomal Abnormalities

**DOI:** 10.1371/journal.pone.0123343

**Published:** 2015-04-29

**Authors:** Ellika Sahlin, Meeli Sirotkina, Andreas Marnerides, Erik Iwarsson, Nikos Papadogiannakis

**Affiliations:** 1 Department of Molecular Medicine and Surgery and Center for Molecular Medicine, Karolinska Institutet, CMM L8:02, Karolinska University Hospital, S-171 76, Stockholm, Sweden; 2 Center for Perinatal Pathology and Department of Pathology, Karolinska University Hospital, Huddinge and Karolinska Institutet, S-141 86, Stockholm, Sweden; Hospital Authority, CHINA

## Abstract

**Objective:**

The biological importance of calcifications occasionally noted in fetal tissues (mainly liver) at autopsy or ultrasound is largely unexplored. Previous reports hint at an association to infection, circulatory compromise, malformations or chromosomal abnormalities. To identify factors associated with calcifications, we have performed a case-control study on the largest cohort of fetuses with calcifications described thus far.

**Methods:**

One-hundred and fifty-one fetuses with calcifications and 302 matched controls were selected from the archives of the Department of Pathology, Karolinska University Hospital. Chromosome analysis by karyotyping or quantitative fluorescence-polymerase chain reaction was performed. Autopsy and placenta reports were scrutinized for presence of malformations and signs of infection.

**Results:**

Calcifications were mainly located in the liver, but also in heart, bowel, and other tissues. Fetuses with calcifications showed a significantly higher proportion of chromosomal abnormalities than controls; 50% vs. 20% (p<0.001). The most frequent aberrations among cases included trisomy 21 (33%), trisomy 18 (22%), and monosomy X (18%). A similar distribution was seen among controls. When comparing cases and controls with chromosomal abnormalities, the cases had a significantly higher prevalence of malformations (95% vs. 77%, p=0.004). Analyzed the other way around, cases with malformations had a significantly higher proportion of chromosomal abnormalities compared with controls, (66% vs. 31%, p<0.001).

**Conclusion:**

The presence of fetal calcifications is associated with high risk of chromosomal abnormality in combination with malformations. Identification of a calcification together with a malformation at autopsy more than doubles the probability of detecting a chromosomal abnormality, compared with identification of a malformation only. We propose that identification of a fetal tissue calcification at autopsy, and potentially also at ultrasound examination, should infer special attention towards co-existence of malformations, as this would be a strong indicator for a chromosomal abnormality.

## Introduction

**Fig 1:**
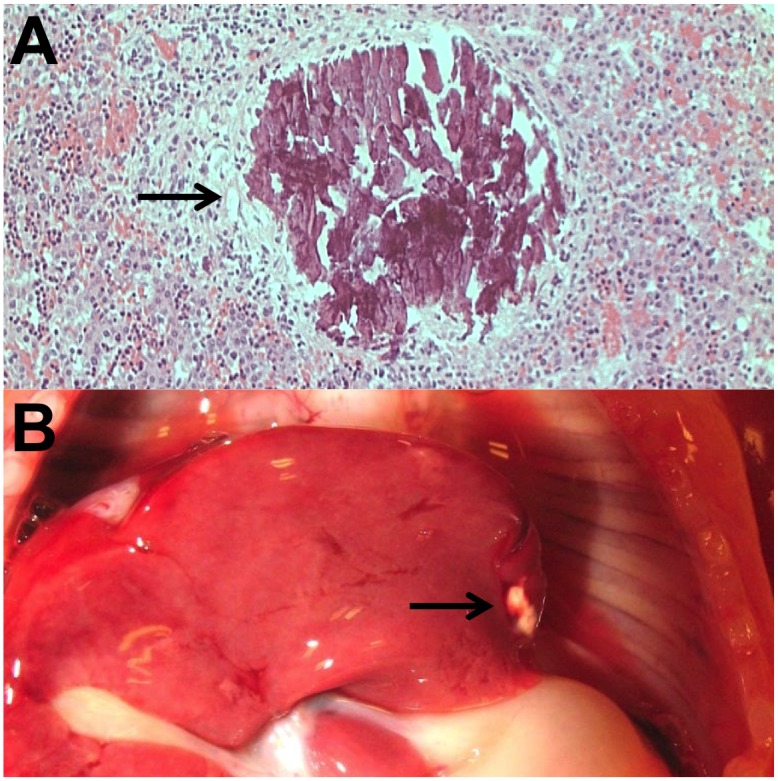
Fetal liver calcification seen on histological section (A) and macroscopically (B). The histological section is stained with Hematoxylin and Eosin, according to standard procedure.

The presence of calcifications in fetal tissues is occasionally recognized both at autopsy and on ultrasound imaging, but their biological importance remains poorly understood. At autopsy, calcifications are identified on histological sections or even macroscopically, if sufficiently large (Fig 1). On ultrasound they are recognized as hyperechogenic sites, which echogenicity resembles that of the surrounding bone [1].

Previous studies have mainly focused on liver calcifications, which have been reported in 2.2% to 4.2% of cases in autopsy studies [2,3], and with an estimated incidence ranging from 1:260 to 1:1750 in ultrasound screening [4–6]. When identified by ultrasound, cases with calcification as the only aberrant finding usually have a good outcome, i.e. the birth of a healthy child [4–10]. However, when identified together with other abnormalities, the prognosis is poor. Studies have suggested association of calcifications with infection [9,11,12], circulatory compromise [2,13], and chromosomal abnormalities [2,4–6,8,9,14].

Fetal liver calcifications have been identified in a number cases of trisomy 18 [5,6,8], as well as in cases of other aneuploidies [2,4,9,14]. Additionally, a high incidence of various chromosomal abnormalities has been identified in fetuses with calcifications located in the heart [15,16]. Taken together, the association between fetal tissue calcifications and chromosomal abnormalities has been indicated in previous studies. Here we explore this association by a matched case-control study.

## Methods

### Study population

The study included 151 fetuses with calcifications and 302 matched controls. The cases were retrospectively identified from the archives of the Center for Perinatal Pathology at the Department of Pathology, Karolinska University Hospital, corresponding to all cases with registered fetal calcifications from January 1, 2003 to December 31, 2012. All histological sections were reexamined by two perinatal pathologists to verify the presence of calcifications. All sections were originally stained with Hematoxylin and Eosin, according to standard procedure. In several dubious cases, special staining (von Kossa) was applied to verify the presence of calcifications. Fetuses from the same archives with the closest analysis date before and after each case, were selected as controls and matched for gestational age (GA) and type of death (spontaneous or missed abortion, stillbirth, induced termination of pregnancy). Missed abortion was defined as fetal death *in utero* (up to gestational week 21+6) that had not been followed by immediate expulsion. Stillbirth was defined as fetal death occurring later than gestational week 22+0. Autopsy and placenta reports for all study subjects were scrutinized with focus on the presence of malformations and signs of infection. Malformation was defined as major structural anomaly in the fetus; for example, minor dysmorphism, isolated abnormal lung lobation, simian crease or simple ectopia of an organ or a tissue was not included. Signs of infection, irrespective of gestational age, were sought for in the placenta (acute chorioamnionitis, vasculitis or funisitis, representing bacterial infection, or chronic villitis, representing viral infection) or the fetus (most often bronchopneumonia). In some cases of stillbirth the infection was corroborated by positive bacterial culture. Viral infection (most notably cytomegalovirus) was in some cases documented by immunohistochemistry or positive viral serology.

### Karyotype and QF-PCR

Chromosome analysis by conventional karyotyping or quantitative fluorescence-polymerase chain reaction (QF-PCR) had previously been performed on 290 of the 453 fetuses included in the study, according to analysis results from the archives of the Clinical Genetics Unit, Karolinska University Hospital. For the remaining fetuses, tissue samples were collected from the biobank of the Department of Perinatal Pathology, Karolinska University Hospital, for complementary analysis by QF-PCR. For cases analyzed by karyotyping, at least 11 metaphase nuclei per sample were analyzed with conventional Q-banding, using standard cytogenetic procedures. In cases where cell culturing was unsuccessful, and for the samples collected retrospectively in the biobank, DNA was extracted from amniocytes, chorionic villi, or fetal tissue using the InstaGene Matrix protocol (Bio-Rad), and analyzed using a QF-PCR panel for detection of aneuploidies involving chromosomes 13, 18, 21, X and Y as previously described [17,18].

### Statistical analysis

McNemar’s test for matched case-control studies was used to determine statistically significant differences between proportions of chromosomal abnormalities and malformations in cases and controls, as well as in subgroups (gestational age intervals, different types of death, and different tissue locations of calcifications). The significance level of all analyses was set to 0.05. However, as each case was matched with two controls, a Bonferroni correction of the significance level was made; hence the significance level was 0.025 in the McNemar calculation. A chi-squared test was used to assess statistically significant differences in distribution of identified chromosomal abnormalities in cases and controls, as well as to detect significant differences in the amount of malformations and signs of infection between the groups. All calculations were performed using IBM Statistical Package for the Social Sciences (SPSS) version 21.

### Ethics

This study was approved by the local ethics committee at Karolinska Institutet (Dnr 2008/670-31/2). All samples were anonymized and de-identified prior to analysis.

## Results

**Fig 2:**
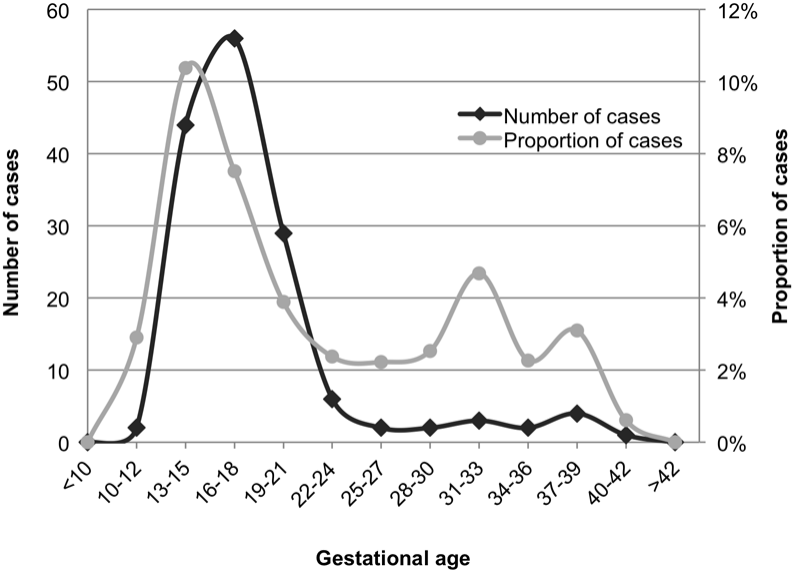
Amount of fetuses with tissue calcifications. The graph illustrates the number and proportion of fetuses with tissue calcifications identified in the archives of the Center for Perinatal Pathology at the Department of Pathology, Karolinska University Hospital, Karolinska University Hospital, 2003–2012.

The overall proportion of fetuses with calcifications in the archives was 5.3%. The proportion showed a steady increase over the years of analysis, from 3.1% in 2003, to 8.2% in 2012. The highest proportion of calcifications was seen among fetuses in gestational week 13–15, where it exceeded 10% (Fig 2). Calcifications were mainly located in the liver (57%), but also in heart (13%), bowel (6%) and other tissues. Calcifications in multiple tissues were identified in 22% of the cases. Fetuses with calcifications showed a significantly higher proportion of chromosomal abnormalities compared with controls, 50% vs. 20% (p<0.001). The proportion of chromosomal abnormalities in all subgroups is summarized in Table 1. For subgroups based on gestational age intervals, the highest proportion of chromosomal abnormalities was seen in cases of gestational age (GA) <14 (71%) and 23–28 (75%), although the number of cases was too low to reach statistical significance in the latter group. The lowest proportion of chromosomal abnormalities was identified in fetuses of GA >29, and no significant difference was detected between cases and controls in this subgroup (17% vs. 13% in cases and controls, respectively). For subgroups based on type of death, the highest proportion of chromosomal abnormalities was detected among cases after induced termination, where both cases and controls had a higher proportion than the average (63% and 34%, respectively). No significant difference was detected between cases and controls in the stillbirth group, and no chromosomal abnormalities at all were detected in the spontaneously aborted fetuses. However, the number of cases was low in both of these subgroups. The tissue location of calcifications did not influence the proportion of chromosomal abnormalities identified.

**Table 1:** Proportion of chromosomal abnormalities and malformations in fetal cases with tissue calcifications, and matched controls.

	Number of cases + controls	Chromosomal abnormalities in cases	Chromosomal abnormalities in controls	P-value	Malformations in cases	Malformations in controls	P-value
Complete cohort	151+ 302	50%	20%	<0.001	72%	50%	<0.001
**Gestational age**							
<14	35+ 70	71%	26%	<0.001	86%	47%	<0.001
15–22	100+ 200	46%	20%	<0.001	68%	54%	0.0011
23–28	4+ 8	75%	0%	0.0412	100%	25%	0.0412
>29	12+ 24	17%	13%	1	48%	29%	0.0961
**Type of death**							
Spontaneous abortion	6+ 12	0%	0%	1	33%	25%	1
Missed abortion	69+ 138	48%	11%	<0.001	55%	27%	<0.001
TOP	60+ 120	63%	34%	<0.001	97%	84%	0.0013
Stillbirth	16+ 32	31%	9%	0.0704	69%	28%	0.0059
**Tissue location of calcifications**							
Liver	85+ 170	51%	21%	<0.001	72%	55%	<0.001
Heart	19+ 38	53%	18%	<0.001	79%	47%	0.006
Bowel	8+ 16	50%	0%	0.0133	50%	31%	0.5050
Multiple	32+ 64	59%	22%	<0.001	75%	41%	<0.001

TOP = termination of pregnancy. The p-values are derived from McNemar’s test for matched case-control studies, and indicate if there are significant differences in the proportions of chromosomal aberrations or malformations between cases and controls. P-values below 0.025 are considered statistically significant.

**Fig 3:**
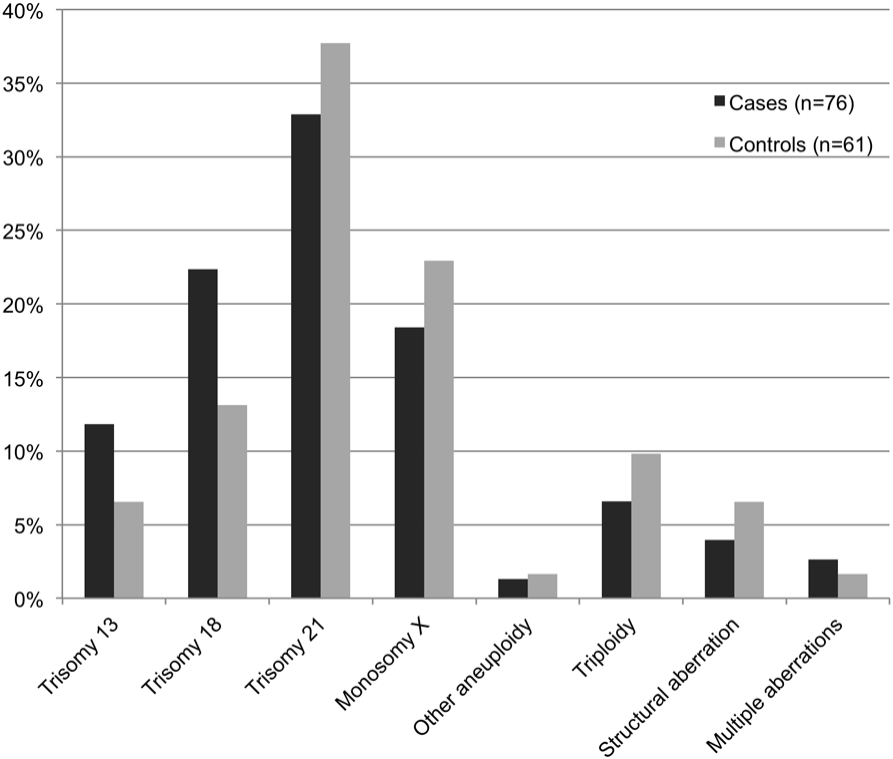
Distribution of chromosomal abnormalities in fetal cases with tissue calcifications and controls. All fetuses were identified in the archives of the Center for Perinatal Pathology at the Department of Pathology, Karolinska University Hospital.

The most frequent chromosomal abnormalities identified included trisomy 21 (33% vs. 38% in cases and controls, respectively), trisomy 18 (22% vs. 13%), monosomy X (18% vs. 23%), and trisomy 13 (12% vs. 7%). The distribution of all aberrations is summarized in Fig 3. Although trisomy 13 and 18 seemed more frequent in cases than in controls, the difference was not statistically significant (p = 0.676). No significant difference in distribution was detected in the subgroups of gestational age intervals, type of death or tissue location of calcifications.

**Fig 4:**
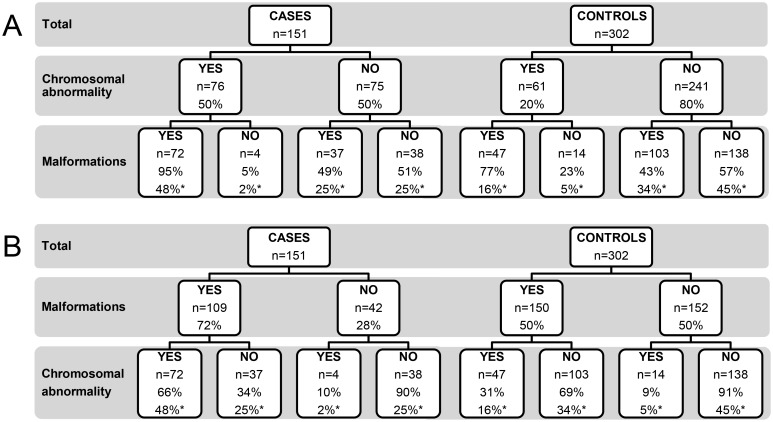
Prevalence of chromosomal abnormalities and malformations in fetal cases with tissue calcifications and controls. (A) Proportion of fetuses with malformations subdivided by the presence of chromosomal abnormalities or not. (B) Proportion of fetuses with chromosomal abnormalities subdivided by the presence of malformations or not. * = percentage of the total number of cases or controls. Percentage without an asterisk refers to the number of subjects in the preceding subdivision.

The prevalence of fetal malformations was significantly higher in cases than in controls (72% vs. 50%, p<0.001). When divided into groups based on the presence of chromosomal abnormalities or not, the difference in malformation prevalence was only seen between cases and controls with chromosomal abnormalities (95% vs. 77%, p = 0.004). The corresponding numbers in cases and controls without chromosomal abnormalities were 49% vs. 43% (p = 0.446). The distribution of malformation prevalence is summarized in Fig 4A. When analyzed the other way around, i.e. the proportion of chromosomal abnormalities in cases and controls with or without malformations, cases with malformations had a significantly higher proportion of chromosomal abnormalities compared with controls, (66% vs. 31%, p<0.001). Cases and controls without malformations showed equal proportions of chromosomal abnormalities (Fig 4B). The proportion of malformations in subgroups is displayed in Table 1.

Data indicating signs of infection were available for 89% of the cases and 88% of the controls. Significantly fewer cases than controls showed signs of infection (10% vs. 18%, p = 0.0378). In the whole cohort, signs of infection were significantly less common in subjects with chromosomal abnormalities than in subjects without (5% vs. 19%, p<0.001).

## Discussion

We describe the first matched case-control study on fetal tissue calcifications, in which we show an association between calcifications and chromosomal abnormalities; 50% vs. 20% in cases and controls, respectively. When creating subgroups based on type of death, the highest proportion of chromosomal abnormalities in both cases and controls was identified in terminated pregnancies (63% and 34%, respectively). This was expected as the main reason for pregnancy termination followed by autopsy is a fetal chromosomal abnormality. The lowest proportion, 31% in cases and 9% in controls, was found in the stillbirth group, except from the subgroup of spontaneously aborted fetuses, where no aberrations were identified. However, as the spontaneous abortion group included only six cases, no conclusions can be drawn. The likely explanation for the low number of chromosomal abnormalities in the stillbirth group is that fetal chromosomal abnormalities is not as frequent after gestational week 22 compared with earlier in pregnancy, as the vast majority of fetuses with chromosomal abnormalities are spontaneously aborted or detected and terminated earlier in pregnancy [19]. This is also reflected in the subgroups based on gestational age; the proportion of chromosomal abnormalities decreases from 71% of cases of GA <14 to 17% in cases of GA >29 (with the exception of fetuses of GA 23–28). The distribution of identified chromosomal abnormalities did not differ significantly between cases and controls. We noted a tendency that trisomy 13 and 18 was more frequent in cases than in controls, but a larger cohort would be required to establish a true difference in distribution.

Malformations were significantly more common in cases compared with controls (72% vs. 50%, p<0.001). When comparing cases and controls with chromosomal abnormalities, the cases showed a significantly higher prevalence of malformations (95% vs. 77%, p = 0.004), while no such difference was detected between cases and controls without chromosomal abnormalities (49% vs. 43%, p = 0.446). It was expected that fetuses with chromosomal abnormalities would have a higher prevalence of malformations than those without, but the discrepancy in prevalence between cases and controls is notable. These results could indicate that calcification is a part of the phenotypic spectrum caused by various chromosomal abnormalities, and is more likely to occur in fetuses with a more severe phenotype. This is in line with a small study on fetal liver calcifications, where Simchen et al. reported that 10 of 11 cases with abnormal karyotypes had malformations that were visible on ultrasound imaging [9], and is supported by our finding that cases without malformations are no more likely than controls without malformations to have a chromosomal abnormality (10% vs 9%, Fig 4B). Chromosomal abnormalities were identified in a significantly higher proportion of cases with malformations compared with controls with malformations (66% vs. 31%, p<0.001). In practice, our results suggest that if a fetus has a calcification and a malformation, the probability of a chromosomal abnormality is increased by more than double, compared with if the fetus has a malformation only.

Signs of infection were identified in significantly fewer cases than controls (10% vs. 18%, p = 0.0378). Previous studies have reported infections in fetuses with liver calcifications [9,11,12], and infections may possibly cause calcifications in individual cases. However, in the present case-control study, we find no support for an association between calcifications and infection.

A limitation of this study is that two different methods were used for chromosome analysis. Among the cases, 48% were analyzed by conventional karyotyping and 52% by QF-PCR. The corresponding numbers among controls were 38% and 62%, respectively. A drawback of QF-PCR is that it only gives information about a limited number of chromosomes, and that no structural aberrations can be detected. In fetuses analyzed by karyotyping, the proportion of detected aberrations was increased by 5.6% and 5.2% in cases and controls, respectively, compared with if the same fetuses would have been analyzed by QF-PCR only. If the complete study cohort had been analyzed by karyotyping, the proportion of detected aberrations could potentially have increased from 50% to 54% among cases and from 20% to 24% among controls, assuming that the 5.6% and 5.2% increase rate would hold true in fetuses analyzed by QF-PCR in our study. Although karyotype analysis of all fetuses most probably would have identified an additional number of chromosomal abnormalities, QF-PCR still shows its great value as a complementary analysis when karyotype is unsuccessful or not suitable, as it has the potential to identify the vast majority of cases with chromosomal abnormalities.

Previous studies have mainly focused on fetal liver calcifications, and the liver was the most common location for calcifications also in our material. Autopsy-based studies have reported a prevalence of liver calcifications of 2.2% to 4.2% of fetuses [2,3]. The corresponding number in our material (isolated liver calcifications and multiple locations including liver) was 4.0%. The overall proportion of calcifications, including all tissues, was 5.3%. We show that calcifications are associated with chromosomal abnormalities regardless of the tissue location of the calcification. The percentage of fetuses with documented calcifications in the archives showed a steady increase over the years 2003–2012. To some extent, this could be influenced by changes in autopsy routines and differences in documentation. However, there was a clear relationship between the increase in identified calcifications and the GA distribution in the archives. During the same time period, 2003–2012, the proportion of fetuses of GA 13–15 in the archives increased at an almost identical rate as the proportion of reported calcifications (S1 Fig). The reason for the increase of fetuses in this gestational age interval is not entirely clear, but is likely related to the introduction of the first trimester combined test in the Stockholm region year 2005. As fetuses in this gestational age interval had the highest prevalence of calcifications (10.4% compared with the overall proportion 5.3%), this is probably the main explanatory factor behind the increase of reported calcifications. The reason why fetuses of this gestational age are more prone to calcifications could possibly be associated with the calcium metabolism in the developing fetus. At gestational week 8–12, skeletal development changes from being completely cartilage-based to the formation of primary ossification centers, which allow bone calcification to begin [20]. The calcification requires a higher calcium concentration, which together with the change in calcium metabolism may lead to an increased vulnerability for tissue calcifications at this time in gestation. However, further research is required to understand the mechanism behind the association between calcifications and chromosomal abnormalities.

## Conclusion

In summary, we have shown that fetal tissue calcifications are highly associated with chromosomal abnormalities in combination with congenital malformations. Identification of a calcification together with a malformation at autopsy more than doubles the probability of detecting a chromosomal abnormality, compared with identification of a malformation only. We propose that identification of a fetal tissue calcification at autopsy, and potentially also at ultrasound examination, should infer special attention towards co-existence of malformations, as this would be a strong indicator for a chromosomal abnormality.

## Supporting Information

S1 Fig Increasing proportions of calcifications and fetuses in gestational age interval 13–15. The proportion of calcifications identified in fetal tissues at the Department of Pathology, Karolinska University Hospital, increased at a similar rate to the frequency of analyzed fetuses of gestational age (GA) 13–15.
(PDF)

S1 Table Complete dataset containing detailed information about all fetuses included in the study. (XLSX)
