# Supplementary material for: Fetal Calcifications Are Associated with Chromosomal Abnormalities
Source: PLoS One. 2015 Apr 29;10(4):e0123343. doi: 10.1371/journal.pone.0123343 (PMC4414523; doi:10.1371/journal.pone.0123343)
Supplement: S1 Fig — The proportion of calcifications identified in fetal tissues at the Department of Pathology, Karolinska University Hospital, increased at a similar rate to the frequency of analyzed fetuses of gestational age (GA) 13–15. (PDF) [file pone.0123343.s001.pdf]

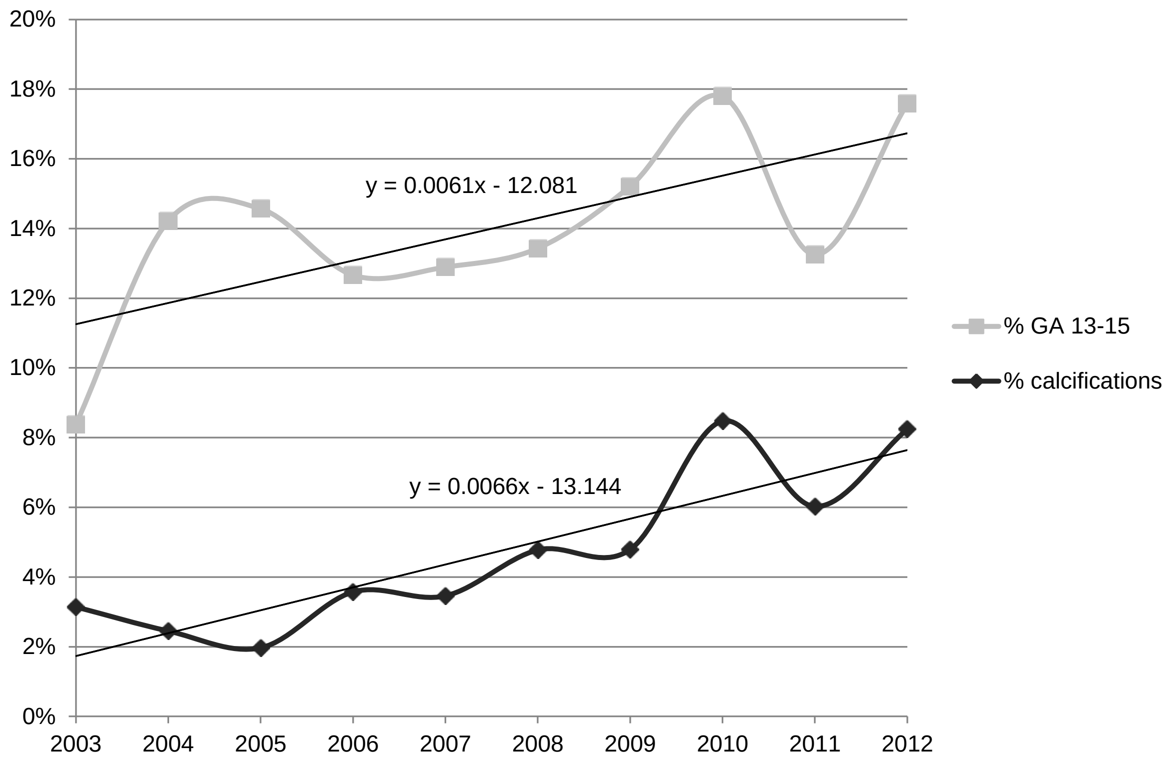

S1 Fig. Increasing proportions of calcifications and gestational age interval 13-15. The proportion of calcifications identified in fetal tissues at the Department of Pathology, Karolinska University Hospital, increased at a similar rate to the frequency of analyzed fetuses of gestational age (GA) 13-15.
